# Bridging chemical space and biological efficacy: advances and challenges in applying generative models in structural modification of natural products

**DOI:** 10.1007/s13659-025-00521-y

**Published:** 2025-06-06

**Authors:** Chuan-Su Liu, Bing-Chao Yan, Han-Dong Sun, Jin-Cai Lu, Pema-Tenzin Puno

**Affiliations:** 1 School of Traditional Chinese Materia Medica, Shenyang Pharmaceutical University, Shenyang, 110016 Liaoning China; 2 State Key Laboratory of Phytochemistry and Natural Medicines, Kunming Institute of Botany, Chinese Academy of Sciences, Kunming, 650201 Yunnan China

**Keywords:** Natural products, Artificial intelligence, Molecular generative models, Structural modification

## Abstract

Natural products (NPs) are invaluable resources for drug discovery, characterized by their intricate scaffolds and diverse bioactivities. AI drug discovery & design (AIDD) has emerged as a transformative approach for the rational structural modification of NPs. This review examines a variety of molecular generation models since 2020, focusing on their potential applications in two primary scenarios of NPs structure modification: modifications when the target is identified and when it remains unidentified. Most of the molecular generative models discussed herein are open-source, and their applicability across different domains and technical feasibility have been evaluated. This evaluation was accomplished by integrating a limited number of research cases and successful practices observed in the molecular optimization of synthetic compounds. Furthermore, the challenges and prospects of employing molecular generation modeling for the structural modification of NPs are discussed.

**Graphical Abstract:**

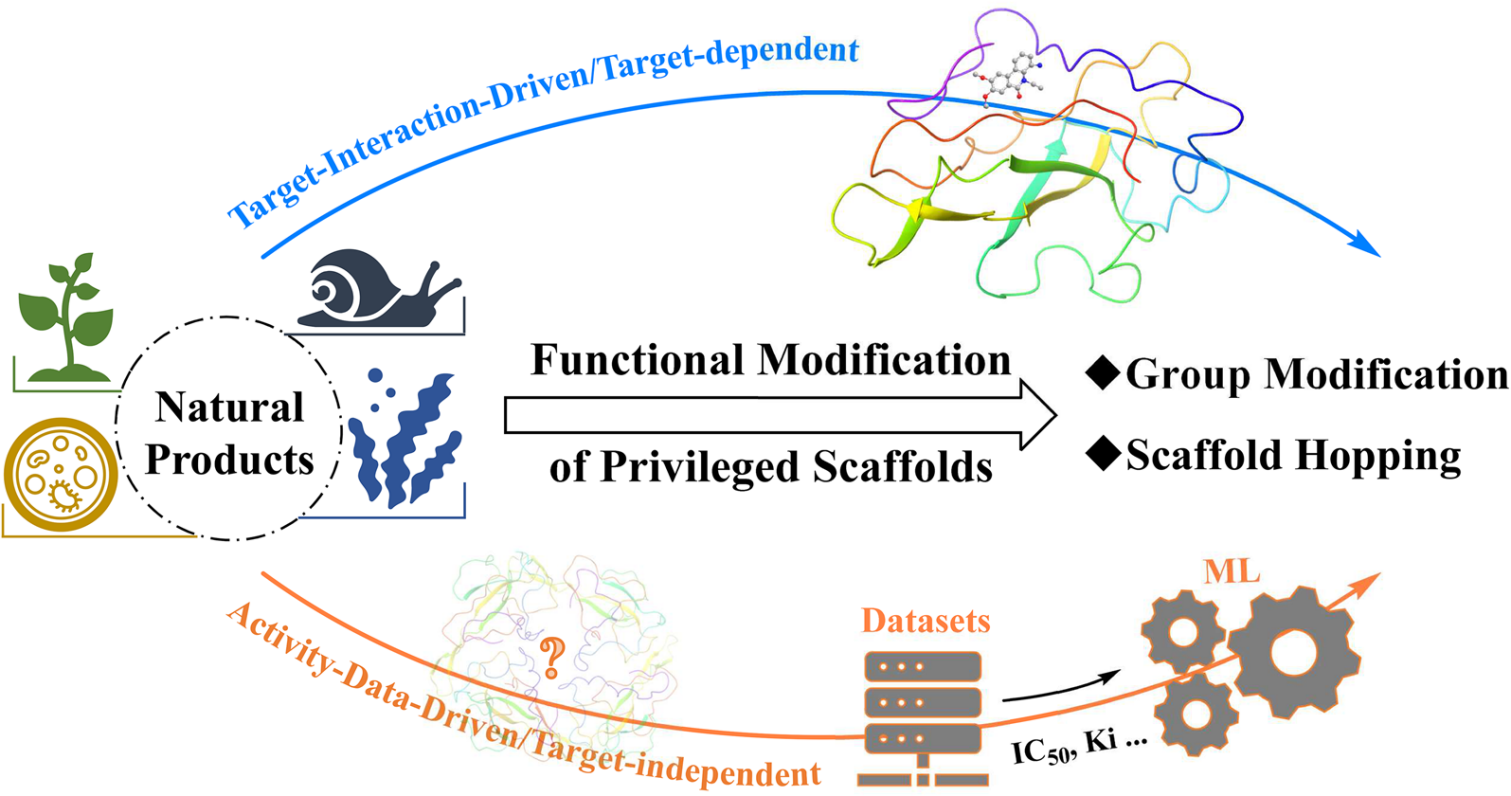

## Introduction

Natural products (NPs) have long been regarded as invaluable resources in drug development, consistently yielding innovative leads and drugs for the treatment of human diseases over the past century [1, 2]. Approximately 30% of FDA-approved drugs from 1981 to 2019 originated from natural products (NPs) or their derivatives (NPDs) [3], particularly in the areas of anti-infectives (e.g., penicillin and vancomycin) and anti-tumors (e.g., paclitaxel and camptothecin) [4, 5]. These secondary metabolites derived from plants [6], animals [7], and microorganisms [8] provide invaluable clues for drug discovery due to their unique chemical scaffolds and evolutionarily optimized bioactivities [9]. Despite their remarkable potential in drug development, the clinical applications of NPs face multiple challenges. Their complex stereochemical structures result in unfavorable ADMET properties and violate Lipinski's rule, which often hinders drug development owing to low intestinal absorption and poor oral bioavailability. In addition, most natural products exhibit certain limitations in terms of biological activity, including low potency, limited specificity, and high toxicity, necessitating structural optimization to enhance the efficacy and selectivity [10–12]. Consequently, overcoming the inherent defects of natural products through structural modification to achieve druggability optimization has become a critical challenge in the field of medicinal chemistry.

Structural modification of NPs predominantly focuses on their core scaffolds. The most commonly used methods include group modification [13], scaffold hopping [14], and structure simplification [15]. Nevertheless, obtaining optimal NPDs continues to pose a significant challenge, even after multiple iterations of structural modifications [10]. Confronted with the significant expenses and inefficiencies of traditional trial-and-error methodologies, as well as the inadequacies of rational design in conventional approaches, computer-aided drug design (CADD) technologies, such as molecular docking, molecular dynamics simulations, and quantitative structure–activity relationship (QSAR) models, have emerged as pivotal tools. These technologies enable the swift evaluation of the affinity of NPDs for target proteins or potential bioactivity, thereby offering more useful guidance for NPs' structural modification [16, 17]. In recent years, with the exponential growth of database scale and breakthroughs in artificial intelligence (AI) algorithms, AIDD has emerged as an evolution of CADD. By fusing generative deep learning with multimodal data, AIDD has realized the fusion of multi-omics data, expansion of generative chemical space, and optimization of dynamic efficacy prediction [18–20]. These advancements are redefining the paradigm of NPs structural modification, transitioning from a “trial-and-error optimization” approach to a “data-driven rational design” strategy. This evolution presents a groundbreaking avenue for addressing the efficiency constraints associated with traditional methods.

Within the AIDD technological framework, molecular generative models, as pivotal methodological advancements, can be primarily categorized into de novo generation and lead optimization. The latter emphasizes directional structural modifications while preserving the core scaffold [21], which methodologically aligns with the central strategy of NPs structural modification “functional modification of privileged scaffolds” [12, 22]. Building on this methodological synergy, this review systematically categorizes molecular generative models developed from 2020 to 2024, focusing on open-source ones. By integrating case studies of their application in the structural modification of bioactive compounds, we critically review advances in leveraging generative models for NPs optimization. In addition, we examine the existing challenges and discuss potential future pathways for interdisciplinary research.

## Classification of molecular generation models

The strategy of “functional modification of privileged scaffolds” encompasses two primary approaches for molecular optimization: group modification and scaffold hopping, which operate at local and global levels, respectively. Group modifications, such as side-chain decoration [12] and fragment replacement [23], are directed at specific local regions of molecules (e.g., the modification site). Side-chain decoration focuses on the alteration of acyclic small groups, whereas fragment replacement emphasizes the substitution of functional fragments. Scaffold hopping [24] is primarily aimed at reconstructing the core scaffold. When the core structure of a molecule is redefined as a connected region, both linker design [25, 26] and scaffold hopping functionally and logically manifest as the directed optimization of the molecule's central connected portion [21].

In the context of the two primary scenarios of “target-known” and “target-unknown” in NPs' structural modification, the models discussed in the review are further subdivided into “target-interaction-driven” and “molecular activity-data-driven” approaches. The former is predominantly applicable to the structural modification of NPs with known target proteins, thereby enhancing the specificity and success rate of drug development [27, 28]. The latter is applicable not only to scenarios where the disease target protein is unknown [29], but also to the discovery of lead compounds and physicochemical optimization of NPs, offering innovative opportunities for the research and development of NPs.

### Models for functional group modification

These models focus on the structural modification of molecular characteristic regions (e.g., side chains and functional groups) to enhance biological activity (e.g., enhancing target interaction) and improve physicochemical properties (e.g., solubility and stability) through “fine-tuning” [12].

#### Target-interaction-driven strategy

This type of models is capable of utilizing protein–ligand interaction data to mine patterns, thereby providing strong support for NP structural modification with known targets [27]. The breakdown for such models focuses on how the generation process interacts and aligns with the target to generate molecules that meet the binding requirements of the target [30] (Table 1).

**Table 1:** Classification of target-molecule interaction driven models

Models	Architecture	Splicing	Growth	Editing	Notes
DeepFrag [31]*	3D DCNN	√			Generation to Classification
FREED [32]*	GCN + RL	√			SAC + PER
FREED++ [33]*	GCN + RL	√			Simplified & Optimized FREED
DEVELOP [34]*	GNN + CNN	√			3D Pharmacophore Characterization
STRIFE [35]*	GNN + CNN	√			FHMs & Dynamic-Extended
POEM [36]*	GGNN	√			Fragment Alignment & Positioning
DrugGPS [37]*	3D Graph XFMR	√			Subpocket Interaction
TACOGFN [38]*	GFlowNet	√			Graph Transformer
PGMG [39]*	GGCN	√			Predefined Fragments
FRAME [40]*	SE(3)-ENN	√			Explicit Interaction Modeling
D3FG [41]*	Diffusion + SE(3)-EGN	√			Rigid Functional Group
AUTODIFF [42]	Diffusion + SE(3)-ECN	√			Conformal Motif Strategy
MolEdit3D [43]	3D Graph	√		√	3D Graph Editing Model
3D-MolGNN_*RL*_ [44]	G-SchNet + RL		√		Critic & Dynamic-Feedback
DiffDec [45]*	Diffusion + E(3)-EGNN		√		Fake Atom Mechanism
AutoFragDiff [46]*	ARDM		√		Dynamic Fragment Generation
PMDM [47]*	Diffusion + SchNet		√		Local & Global Diffusion Strategy
DeepICL [48]*	VAE + E(3)-IIN		√		E(3)-Invariant Interaction Network
DiffInt [49]*	E(3)-Conditional Diffusion		√		Hydrogen-Bonded Particles
TargetSA [50]*	SA Algorithm		√	√	Adaptive Simulated Annealing
Delete [51]*	Unified Masking		√	√	Local Editing Model
DrugHIVE [52]*	HVAE		√		Prior-Posterior Sampling

The symbol [*] indicates that the model is an open-source model

##### Fragment splicing methods

**Fig. 1:**
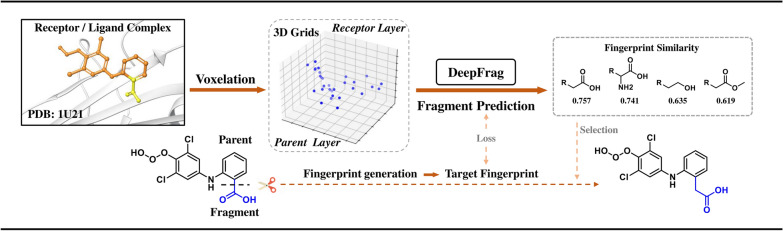
DeepFrag workflow: The “Parent” and “Fragment” of the initial ligand are highlighted in orange and yellow respectively, which are converted into 3D voxel grids (density channel), merged and fed into the DeepFrag model to predict the missing fragment fingerprints. Finally, the generated fragment fingerprints were compared with a predefined fingerprint library to obtain the prediction results.

These models select fragments from a predefined chemical fragment library (e.g., pharmacophores and functional groups) and splice them onto the scaffold to generate molecules, ensuring the chemical authenticity and synthesizability of the generated molecules [53, 54]. DeepFrag addresses the challenge of molecule generation by transforming it into a classification task. This is achieved by removing a ligand fragment from a protein–ligand complex and querying the machine learning model to determine the appropriate fragment to be inserted in its place. This allows the model to generate the molecule while considering the surrounding receptor pocket and the full ligand molecule [31] (Fig. 1). Building on fragment generation, FREED combines reinforcement learning (RL) and prioritized experience replay (PER) technology to effectively explore the chemical space and generate pharmacochemically acceptable molecules with high docking scores [32]. The subsequent development of FREED++ fixed and improved the original FREED, resolving multiple implementation errors [33]. DEVELOP combines a graph neural network (GNN) and convolutional neural network (CNN) to construct molecular maps bond-by-bond, and partially constrains the dynamics of molecule generation by utilizing 3D pharmacophore information [34]. STRIFE adopts an architecture similar to that of DEVELOP. It dynamically guides the starting molecule to expand pharmacophore fragments complementary to the target by extracting fragment hotspot maps (FHMs) from the protein target [35]. POEM uses a computer vision method to align protein pockets with PDB-derived images for fragment filtering and linking via DeLinker [36]. DrugGPS learns subpocket prototypes and constructs a global interaction graph to guide the selection of the most suitable fragments from the motif library for addition to the generated molecules [37]. TACOGFN incorporates target pocket information into a generative flow network (GFlowNet) and uses a graph transformer to predict docking scores, generating molecules by gradually adding predefined chemical fragments (72 types) and setting connection points [38]. PGMG uses pharmacophores containing chemical features and spatial distribution as the core template, and introduces latent variables to model many-to-many mapping relationships between pharmacophores and molecules to enhance diversity while maintaining target suitability [39]. FRAME utilizes SE(3)-equivariant neural networks to capture the 3D information of target pockets, explicitly model protein–ligand interactions (e.g., hydrogen bonds and π-π stacking), and dynamically select the optimal connection points and fragments from the starting molecule [40]. D3FG defines molecular fragments as rigid functional groups to preserve the structure of complex fragments, predicts connected fragments by capturing spatial relationships and interactions between proteins and ligands via GNN and uses diffusion modeling to determine fragment position and orientation [41]. AUTODIFF proposes a novel conformal motif strategy to preserve the conformational information of local structures by adding redundant virtual atoms at molecular bond breakage sites [42]. MolEdit3D is a 3D graph-editing model that constructs molecules by stepwise addition or deletion of rigid fragments within the target binding site [43]. These fragment splicing methods, based on different frameworks, utilize protein–ligand interaction information to varying degrees and have successfully completed structure-derived tasks. However, the generated molecules are often limited by the fragment database and cannot fully explore the chemical space of the target binding pocket.

##### Molecular growth methods

These models generate molecules directly in the 3D space of the target pocket, maximizing the exploration of the pocket's spatial and target interactions through atom-by-atom or substructure autoregressive generation or global generation based on a diffusion model. 3D-MolGNN_*RL*_ combines RL with a 3D-Scaffold generative model. Starting from specific 3D scaffolds, the model gradually assesses the binding probability and affinity of generated molecules with the target protein, thus dynamically adjusting the generation process [44]. DiffDec directly models the 3D interactions of molecules with the protein pocket, ensuring that the position and orientation of the generated R motifs are complementary to the pocket shape. The model employs a fake atom mechanism to achieve end-to-end flexible generation of R groups of different sizes within the diffusion model [45]. AutoFragDiff predicts the atomic type and spatial coordinates of new molecular fragments, thus dynamically generating fragments to enhance the local geometric accuracy and binding affinity of the generated molecules [46]. PMDM combines local (simulating covalent bonds) and global molecular dynamics (simulating van der Waals forces), as well as a dual diffusion strategy, to generate 3D molecules that fit a specific target pocket [47]. DeepICL utilizes prior knowledge of protein–ligand interactions to achieve interaction-guided atom-by-atom generation of molecules within the binding pocket [48]. DiffInt introduces interaction particles to explicitly handle hydrogen bond interactions, and uses an E(3)-conditional diffusion model to generate ligand molecules forming hydrogen bonds with the target pocket [49]. TargetSA employs an adaptive simulated annealing (SA) algorithm combined with multi-constraint optimization objectives (e.g., binding affinity, drug-like properties, and synthesizability) to iteratively modify the molecular structure through dynamic local editing (insertion, replacement, deletion, and cyclization) to globally search for the optimal result in a discrete chemical space [50]. Delete (Deep lead optimization enveloped in protein pocket) employs a unified deletion (masking) strategy to dynamically mask atoms or molecular fragments, and then combines them with the geometric features of protein pockets for repair generation [51]. DrugHIVE uses a hierarchical variational autoencoder (HVAE), combining molecular and protein density grid information to encode multi-scale spatial features (e.g., atom type and hydrogen bond donor/acceptor) for modeling the protein pocket, and achieves lead optimization tasks through spatial prior-posterior sampling [52]. These models explore the chemical space within the protein target pocket using different strategies developed without relying on existing fragment libraries. However, the synthetic accessibility of the generated molecules must be verified through wet experiments.

#### Activity-data-driven strategy

Examining the structural and activity features of these molecules, which are derived from a recognized dataset of active molecules, enables a thorough investigation of the relationship between the activity and structure [29]. The model predicts the potential activity of NPs by analyzing the common features of active molecules, thereby guiding the structural modification of NPs. The flexibility of the model also enables the exploration of a wider range of chemical spaces and biological activities. For the sub-categorization of these models, more attention is paid to how data are input and processed in the model, and how different types of data inputs affect the learning and generative effects of the model (Table 2).

**Table 2:** Classification of molecular activity-data-driven models

Models	Architecture	SMILES	Graph	3D	Notes
Scaffold Decorator [55]*	RNN	√			Data Augmentation
LibINVENT [56]*	RNN + RL	√			Reaction-Based Slicing
SAMOA [57]*	RNN + RL	√			Flexible Sampling
REINVENT 4 [58]*	RNN + TL + RL + CL	√			Lead Optimization Platform
SAFE [59]*	GPT2-like XFMR	√			A New Linear Representation
MolGPT [60]*	GPT XFMR	√			First XFMR Decoder Architecture
EMPIRE [61]*	VAE	√			Arbitrary Chemical Space
Sc2Mol [62]*	VAE + XFMR	√			Two-Step Generation Strategy
GraphScaffold [63]*	VAE + GNN		√		Multi-Property Control Generation
DrugEx v3 [64]*	Graph XFMR + RL		√		Multi-Objective Constrained
Tree-Invent [65]*	GNN + RL		√		Topological Tree Constrained
MoLeR [66]*	VAE + GNN		√		Motif-Based Generation
MRGVAE [67]*	VAE + MPNNs		√		Molecular Hierarchization
3D-Scaffold [68]*	G-SchNet			√	Directly Output 3D Coordinates
3D-SMGE [69]*	G-SchNet			√	ADMET Optimization

The symbol [*] indicates that the model is an open-source model

##### SMILES sequence-based methods

These models leverage the robust representational capabilities of natural language models (e.g., transformer), facilitating the rapid generation of molecules and demonstrating their suitability for large-scale virtual screening and other application scenarios. However, this model faces illegal issues when multiple side chains are added to the scaffold. To address this problem, the Scaffold Decorator effectively avoids illegal linkages by defining special connection point markers [*] in the SMILES string of the starting scaffold. During the generation phase, the language model grows fragments at these markers in SMILES format, effectively avoiding illegal connections and ensuring that the generated molecular structures are legal and comply with chemical rules. The model provides a data augmentation method that preprocesses all molecules in a small dataset by cutting acyclic bonds (or bonds that comply with RECAP rules), generating a large number of scaffold-decoration tuples and significantly expanding the training data [55]. LibINVENT adopts the same molecular generation strategy as Scaffold Decorator. The model uses a reaction-based preprocessing fragmentation algorithm and combines reinforcement learning (QSAR, ROCS) and a prior model to ensure that the generated compounds adhere to the defined chemical reaction and maintain diversity [56]. SAMOA modifies the recurrent neural network (RNN) sampling process, directly enforcing molecules to follow a predefined scaffold during generation, and uses reinforcement learning (QSAR) to optimize the pharmacological activity and pharmacokinetic properties of the generated molecules [57]. REINVENT 4 trains pre-trained models on large public datasets such as ChEMBL and PubChem. Transfer learning (TL) facilitates the concentration on tasks involving small datasets, while reinforcement learning (RL) and course learning (CL) empower the model to generate molecules with specific properties, thereby progressively enhancing the efficiency and quality of molecular generation [58]. SMILES-based models benefit from their concise text format, which facilitates the storage and transmission of molecular structure data but are also limited by their strict syntactic rules. SAFE proposes a new molecular linear representation method that represents SMILES strings as unordered sequences of interconnected fragment blocks, effectively bypassing complex decoding schemes and simplifying the generation task. The SAFE-GPT model was trained on a dataset containing 1.1 billion SAFE representations and can perform target-oriented lead compound optimization [59].

##### Molecular graph-based methods

These models represent molecules as graph structures with nodes as atoms and edges as chemical bonds, capturing interatomic bonding relationships directly and avoiding the limitations of the SMILES syntax. GraphScaffold starts from a given molecular scaffold and extends it by sequentially adding atoms and bonds. The model can control molecular properties through a conditional generation process [63]. DrugEx v3 combines a graph transformer and RL to process molecular structure information more effectively. The model can deal with more complex scaffold structures and simultaneously optimize multiple objectives (e.g., QED and affinity) [64]. Tree-Invent combines molecular graph topological trees with RL. Model uses three independent sub-models to perform Nodeadd, Ringgen, and Nodeconn operations. This approach achieves precise control over the molecular generation process and optimizes the results according to target properties [65].

##### 3D structure-based methods

These models combine atomic coordinates and chemical bonding information to generate molecules with spatial conformations, effectively addressing the lack of conformational changes in representations based on SMILES and molecular graphs. 3D-Scaffold uses a feature learning module to extract rotation and translation-invariant atomic features, capturing the incomplete chemical environment. The atomic placement module then predicts the type of the next atom (discrete distribution) and its 3D distance distribution with all placed atoms, considering the dynamic interactions in molecular generation and avoiding the molecular rigidity assumption [68]. This type of models relies on high-quality 3D structural data and requires to process multi-modal data involving continuous spatial coordinates and discrete atomic types, resulting in extremely high computational complexity.

From the performance verification experiments of these models, it is evident that activity-data-driven molecular generation models exhibit a certain degree of activity-oriented nature. For instance, Scaffold Decorator, when applied to the DRD2 active molecule set (4,211 molecules), generated molecules from different scaffolds that had a significantly higher proportion of predicted activity (based on a pre-trained activity prediction model) compared to random decoration using the ChEMBL dataset [55]. SAMOA on the MMP-12 inhibitor lead optimization dataset, 23% of the molecules generated by the model met the constrained scaffold and high activity (pre-trained QSAR predicts pIC_50_ > 7.5) [57]. For REINVENT 4, in the task of generating new PDK1 inhibitors, the proportion of active molecules generated through RL was 1.9% (with a docking score ≤ −8 kcal/mol and QED ≥ 0.7). After using TL-RL, this proportion increased to 3.5% (the proportion of active molecules based on RL and RL-TL increased with the number of model runs) [58]. DrugEx v3 carried out targeted molecular generation for A2AAR, with 86.1% of the generated molecules predicted to be active [64]. 3D-Scaffold targeted NSP15 and generated multiple molecules based on Exebryl-1, which showed good binding affinity in docking simulations and had lower SA scores and higher QED scores [68].

##### Other methods

The following models focus on scaffold constraints and the optimization of physicochemical properties. These models are trained on publicly available large datasets to enhance the diversity of molecular generation results and explore a wider chemical space, making them more suitable for lead compound discovery.

**Fig. 2:**
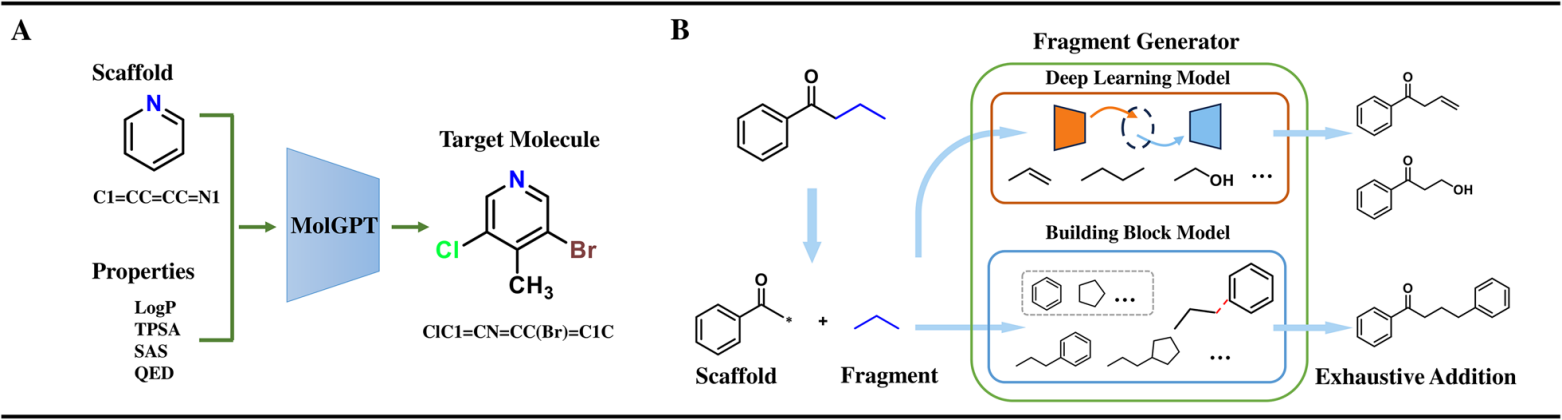
Two representative data-driven methods. **A** MolGPT illustration and **B** EMPIRE illustration

**SMILES-based models.** MolGPT learns the SMILES representation and structural features in molecular datasets (MOSES and GuacaMol) through a masked self-attention mechanism, enabling it to perform conditional generation and control molecular properties (e.g., Log P, TPSA, SAS, and QED) [60] (Fig. 2A). EMPIRE generates new fragments through VAE and a building block list, resulting in novel molecules in arbitrary chemical spaces. For example, EMPIRE can generate molecules containing unique scaffolds (e.g., bicyclo[1.1.1]pentane and cubane) or elements (e.g., boron and silicon), which facilitates virtual screening in unexplored chemical spaces and enhances the efficiency of drug design [61] (Fig. 2B). Sc2Mol decomposes molecular generation into two steps: scaffold generation and scaffold decoration, which are handled by the VAE and transformer, respectively. The model learns the structural features of molecules from molecular datasets (MOSES and ZINC-250 k) and supports the generation of molecules with specific properties from a given scaffold [62].

**Molecular graph-based models.** MoLeR adds atoms or predefined motifs to a complete scaffold with single bonds under strict scaffold-constrained conditions. The model combines the molecular group optimization (MSO) method and can generate molecules with target properties [66]. MRGVAE decomposes molecules in the dataset (ChEMBL) into small fragments and groups them into interchangeable fragment clusters based on the local structural environment of the fragments. The model combines random sampling and top-p sampling to increase fragment diversity while controlling the distribution of chemical properties. The model controls the generation of multiple molecular attributes, such as scaffold structure, molecular weight, and log P, through conditional generation [67].

**3D structure-based models.** 3D-SMGE generates 3D molecular structures from a specific scaffold and evaluates their ADMET properties by learning molecular structures and properties from a large molecular dataset [69].

### Models for scaffold hopping

Local group modification can enhance functional adaptability, but is limited by the inherent defects of the original scaffold (e.g., non-modifiable metabolic sites). Therefore, scaffold hopping is required to achieve a leap in drug-like properties. These models mainly involve global adjustments to the core scaffold or connecting parts of active compounds to optimize their structure and function, thereby breaking through the activity ceiling of the original scaffold [15] (Table 3).

**Table 3:** Classification of scaffold hopping methods

Methods	Architecture	Scaffold Hopping	Linker Design	Notes
Target-interaction-driven				
DeepHop [70]*	3D GNN + XFMR	√		3D Structural Similarity
GraphGMVAE [71]	GMVAE + DualMPNN	√		Side-Chain Retention
ScaffoldGVAE [72]*	VAE + MVGNN	√		Interaction Constraints
DiffHopp [73]*	E(3)-E Graph Diffusion	√		PDBbind Data Learning
DiffLinker [74]*	E(3)-E Diffusion	√	√	Geometric Constraints
Activity-data-driven				
DeLinker [75]*	GGNN	√	√	Distance & Direction
SyntaLinker [76]*	XFMR	√	√	NLP Task
SyntaLinker-Hybrid[77]*	XFMR + TL	√	√	Fragments Hybridization
DRLinker [78]*	XFMR + RL	√	√	RL-Based Scoring
GRELinker [79]*	GGNN + RL + CL	√	√	Outperforms DRlinker
Link-INVENT [80]*	RNN + RL	√	√	Fragment Splicing

The symbol [*] indicates that the model is an open-source model

#### Target-interaction-driven strategy

**Fig. 3:**
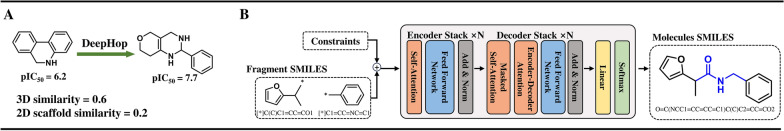
Two representative scaffold hopping methods. **A** DeepHop illustration and **B** SyntaLinker illustration

Focusing on scaffold-hopping molecular generation models, DeepHop integrates the sequence information of the target protein and the 3D conformation information of molecules. This allows the model to generate molecules with similar 3D structures but different 2D scaffolds while considering the target protein information. However, the side chains that need to be retained may be modified in the process [70] (Fig. 3A). To address this limitation, GraphGMVAE inputs the bioactivity condition vector of the protein target together with the scaffold and side-chain embeddings of the molecule into the generative model; however, the GraphGMVAE model is not open-source, which limits its widespread application [71]. The open-source model ScaffoldGVAE, trained on the ChEMBL database and focusing on specific kinase protein data, maps scaffold embeddings to a Gaussian mixture distribution while keeping side-chain embeddings unchanged. This enables the generation and optimization of scaffolds [72]. DiffHopp guides scaffold hopping in a given protein pocket by learning the interaction information of a large number of protein–ligand complexes in PDBbind [73]. DiffLinker is a target-driven linker design model that uses the atomic point clouds of the protein pocket as fixed information and inputs them into the neural network along with the atomic point clouds of the molecular fragment. As a result, the generated molecules can consider the geometric constraints of the protein pocket, thus generating molecules compatible with the pocket structure [74].

#### Activity-data-driven strategy

All the models in this category are linker design models that also take into account scaffold hopping. As the first linker design model to incorporate 3D structural information into the generation process, DeLinker learns the molecular structure and property information from the ZINC and CASF datasets. The model generates molecules by utilizing the relative distance and orientation information between two fragments or partial structures [75]. Benefiting from the expressive power of transformer, SyntaLinker transforms the fragment linking task into a NLP-like task, by parsing the syntactic patterns and fragment linking rules of molecular SMILES sequences in the database and introducing a multi-conditional control mechanism (e.g., the shortest linking distance, the presence or absence of hydrogen bond donors, acceptors, rotatable bonds, and loops).This allows for end-to-end controllable generation from a given fragment pair to a complete molecule [76] (Fig. 3B). Subsequently, SyntaLinker-Hybrid was developed based on the generative model of SyntaLinker, which uses TL to fine-tune target-focused active compound data and combines fragment hybridization technology to generate molecules with target specificity [77]. DRlinker combines transformer and RL to generate compounds under the guidance of a given scoring function and can control specific properties of the generated compounds, such as linker length, log P value, bioactivity, QED, SAS, 3D similarity, and 2D structural novelty [78]. Compared with DRlinker, GRELinker combines the gated graph neural network (GGNN) architecture with RL and CL algorithms. The model has increased the percentage of molecules that meet property constraints and is able to generate more complex linker structures [79]. Link-INVENT is an extension of the existing de novo molecular design platform REINVENT [81] and is specialized for tasks such as fragment linking, scaffold hopping, and PROTAC design. The model is trained on a large number of molecular datasets (e.g., the ChEMBL database) and controls multiple attributes of generated molecules, such as scaffold structure, molecular weight, and log P, through conditional generation [80].

## Application cases of structural modification

### Group modification cases

Local group modification models optimize target affinity by fine-tuning side chains or functional groups while retaining the core scaffold. The following cases demonstrate their applications in fields such as antiviral and anticancer research.

#### Cases of target-interaction-driven generation

##### DeepFrag for target optimization in antiviral drug development

**Fig. 4:**
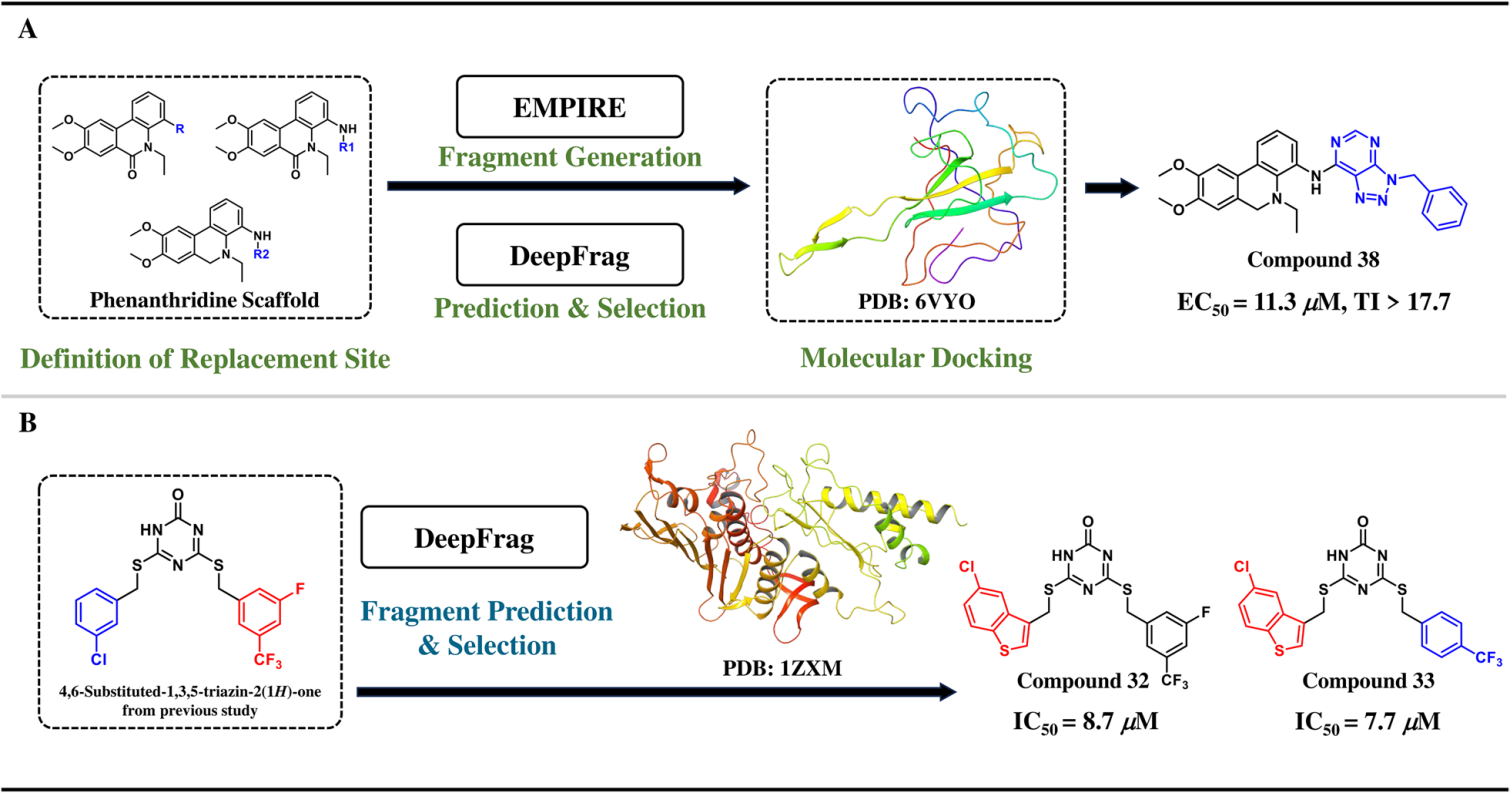
Two application examples of Deepfrag. **A** Antiviral drug development and **B** Topo IIα inhibitors optimization

SARS-CoV-2 N protein is a key target for antiviral drug development [82]. Hao et al. identified the antiviral activity of phenanthridine derivatives through CADD design validation [83] and identified the binding site as the N-terminal domain of the N protein. To obtain lead compounds with higher affinity for N protein and stronger antiviral activity, researchers further employed the DeepFrag model (Sect. 2.1.1) to perform directed replacement of the side chains of phenanthridine alkaloids with antiviral potential based on the local environment of the N protein binding pocket. To balance target-orientedness and diversity, the EMPIRE model (Sect. 2.1.2) was introduced to enhance the generative chemical space. The models generated 16,689 virtual derivatives, and 44 compounds were synthesized after virtual screening and molecular docking. Compound **38** exhibited high affinity and significant antiviral activity in vitro (EC_50_ = 11.3 μM, TI > 17.7) [84] (Fig. 4A).

##### DeepFrag for optimization of anticancer drug Topo IIα inhibitors

Human DNA topoisomerase IIα (topo IIα) is an important target for anticancer drugs [85]. In a previous work by Perdih et al. 4,6-substituted-1,3,5-triazin-2(1*H*)-ones with strong inhibitory activity against topo IIα were discovered through a combination of structure-based and ligand-based pharmacophore models and molecular docking calculations [86]. To enhance their binding affinity and inhibitory activity, researchers used DeepFrag to analyze the ATP-binding site of top IIα and guide the optimization of the R group of the triazine compounds. They also used molecular docking and molecular dynamics (MD) simulations to screen and synthesize 44 derivatives. Compounds **32** and **33** showed a significant increase in inhibitory activity against topo IIα (IC_50_ = 8.7 μM and 7.7 μM, respectively), and exhibited strong cytotoxicity in glioblastoma and breast cancer cell lines [87] (Fig. 4B).

#### Cases for activity-data-driven generation

##### Scaffold Decorator for the discovery of selective antagonists of adenosine A_2B_ receptors

**Fig. 5:**
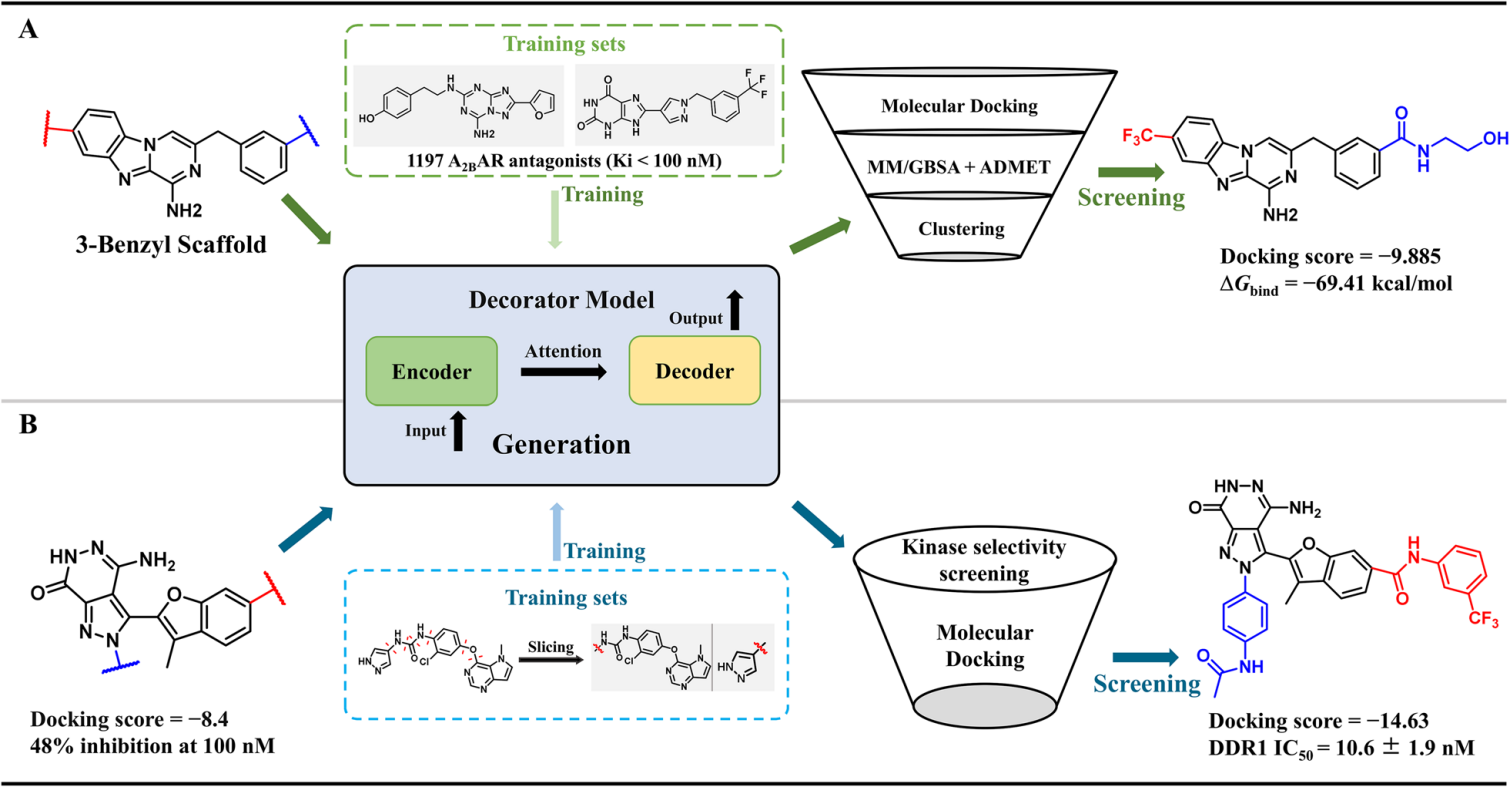
Two application examples of Scaffold Decorator. **A** Discovery of A_2B_ receptors and **B** DDR1 inhibitors

The adenosine A_2B_ receptor (A_2B_AR) is associated with inflammatory diseases, but the development of its antagonists often fails due to poor subtype selectivity [88]. Lei et al. used an activity-data-driven Scaffold Decorator model (Sect. 2.1.2), based on a dataset of known active molecules, to diverse derivatizations of the 3-amine and 3-benzyl scaffold. They generated over 90,000 virtual molecules and used virtual screening, ADMET screening, and QSAR prediction to identify the lead compound **ABA-1266**, which was screened for its free energy of binding to A_2B_AR of −69.41 kcal/mol and showed high selectivity among multiple adenosine receptor subtypes [89] (Fig. 5A).

##### Scaffold Decorator for the discovery of DDR1 selective inhibitors

The receptor tyrosine kinase DDR1 plays a key role in cell signaling, tissue development, and disease progression. However, the development of DDR1 inhibitors is often limited by their poor target specificity [90]. Zheng et al. found that **DC-1**, discovered in previous studies, has inhibitory activity against DDR1 (inhibition rate of 48%). Molecular docking analyses have revealed the potential for optimization of the binding model of **DC-1** and DDR1 [91]. To obtain compounds with high activity specificity, researchers have integrated Scaffold Decorator molecular generation, kinase selectivity screening, and molecular docking to identify a novel DDR1 inhibitor, compound **2**. The compound exhibited excellent selectivity (S(10) = 0.002 out of 430 kinases) with an IC_50_ value of 10.6 ± 1.9 nM for DDR1 and significantly inhibited pro-inflammatory cytokines and DDR1 autophosphorylation [92] (Fig. 5B).

##### LibINVENT for the discovery of new inhibitors of Cbl-b

The E3 ubiquitin ligase Cbl-b plays multiple roles in immune and tumor cells. Inhibiting its function can enhance the antitumor capacity of the immune system and suppress the growth and survival of tumor cells [93]. To identify new Cbl-b inhibitors, Hughes et al. utilized the LibINVENT model (Sect. 2.1.2) with a triazolone core scaffold and optimized pharmacophore matching (ROCS score) and drug-like properties (QED > 0.6) through RL. Through virtual screening and ADMET property prediction, the researchers selected **VC1** and **VC2**, which exhibited good target selectivity. Further structural optimization and in-vitro validation revealed that compounds **20** and **24** had IC_50_ values of 1.4 μM and 0.17 μM, respectively, and demonstrated significant cytotoxicity in various human cancer cell lines [94].

##### SAMOA for dynamic optimization of ATM kinase inhibitors

ATM kinase is a key target of the DNA damage repair pathway and its inhibitors have important potential in anticancer therapy [95]. Chen et al. proposed a dual-modal optimization strategy, utilizing the flexible framework of the SAMOA algorithm (Sect. 2.1.2). Based on a RL process with a composite scoring function of target specificity (SBDD) and ligand similarity (LBDD), 20,194 virtual molecules were generated from the 6-(pyridin-3-yl) quinoxaline scaffold. After molecular docking and ADMET prediction, lead compound **7a** was selected, which exhibited an IC_50_ value of 5 nM for ATM inhibition in vitro but had poor metabolic stability. By replacing different urea groups, compound **8d** (IC_50_ = 2 nM) exhibited better ATM inhibitory activity and partial metabolic stability. Further optimization led to compound **10r**, which showed high selectivity with an IC_50_ value of 1 nM for ATM kinase, almost no inhibitory effect on 103 other kinases (showing high selectivity), and excellent metabolic stability and cellular activity in vitro [96].

### Scaffold hopping cases

Scaffold hopping models overcome the limitations of the original scaffold. The following cases demonstrate the application of these methods in the development of kinase inhibitors.

#### Target-interaction-driven generation for scaffold hopping in JAK1 inhibitors

**Fig. 6:**
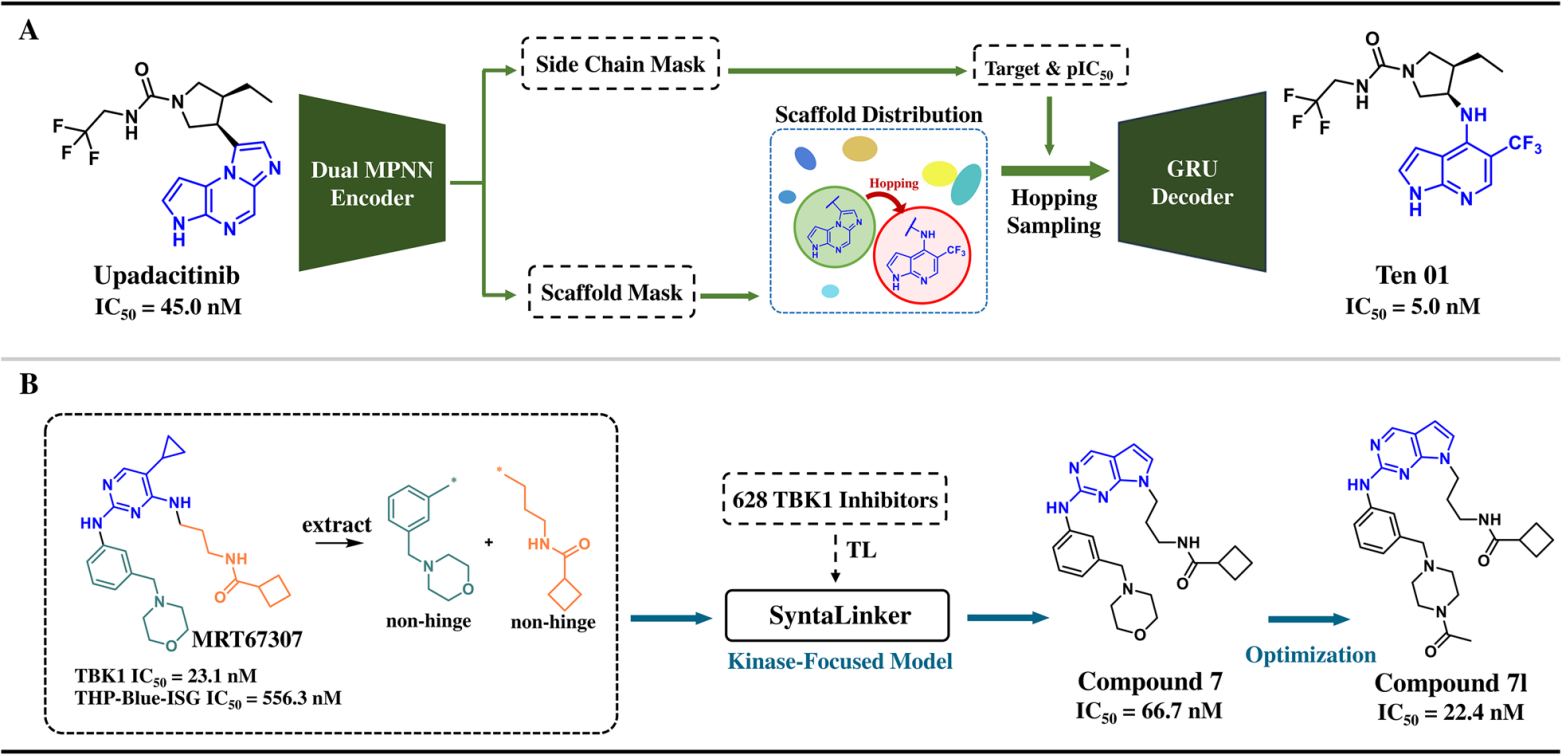
**A** Scaffold hopping of JAK1 inhibitors and **B** linker optimization of TBK1 inhibitors

The non-receptor tyrosine kinase JAK1 plays a key role in cell signaling in immune responses and inflammatory processes [97]. The development of new JAK inhibitors remains highly focused on improving subtype selectivity and enhancing therapeutic effects while reducing toxicity. Huang et al. developed the GraphGMVAE model (Sect. 2.2.1) to generate novel JAK1 inhibitors for validation starting from the FDA-approved upadacitinib. Among the generated molecules, 97.9% had novel scaffolds that differed from those of from known JAK1 inhibitors. Seven compounds were synthesized, with compound **Ten01** showing an IC_50_ value of 5.0 nM for JAK1 inhibition [71] (Fig. 6A).

#### Activity-data-driven generation for linker optimization in TBK1 inhibitors

TANK-binding kinase 1 (TBK1) plays an important role in the innate immune system and is involved in the development of several cancers [98]. Although several small-molecule TBK1 inhibitors have been reported, none have been used clinically. Lu et al. first trained a prior model on a general ChEMBL fragment set and then performed TL on a kinase inhibitor dataset to construct a kinase-specific SyntaLinker model (Sect. 2.2.2). Researchers generated a series of molecules by replacing the non-hinge binding fragments of the known TBK1 inhibitor **MRT67307** and selected lead compound** 7** through molecular docking. Compound **7** exhibited an IC_50_ value of 66.7 nM for TBK1 inhibition and demonstrated good target selectivity. Further structural optimization and in vitro validation led to the identification of compound **7l**, which exhibited an IC_50_ value of 22.4 nM for TBK1 inhibition and significantly inhibited the expression of TBK1 downstream genes in THP1 cells [99] (Fig. 6B).

### Discussion

The limitations in the application of the model are analyzed in relation to the application cases and the classification of the models in Sect. 2.

Firstly, DeepFrag matches fragments from a predefined fragment library and is unable to generate new fragments. It also assumes that fragment addition will not significantly change the binding geometry of the parent molecule, without considering the dynamic changes between the molecule and the binding pocket. In contrast, the pocket-based molecular growth methods (e.g., DiffDec [45], AutoFragDiff [46], and DiffInt [49]) demonstrate the ability to handle interactions with pocket during the molecular generation process, effectively avoiding the limitations of fragment library and the assumption of molecular rigidity.

Secondly, although the activity-data-driven SAMOA model integrates the 3D structural information of ATM kinase through RL, its SBDD module is based on a static crystal conformation and does not simulate the dynamic conformational effects of proteins. Molecular generation models for dynamic interaction modeling (e.g., 3D-MoLGNNRL [44], FRAME [40], and AutoDiff [42]) achieve dynamic control of the molecular generation process based on different architectures, overcoming the limitations of static target modeling and the absence of dynamic interactions.

Thirdly, the above cases of molecular generation methods heavily rely on CADD virtual screening for activity validation, resulting in a disconnect between the generation and validation stages and failing to achieve end-to-end dynamic optimization. For example, Scaffold Decorator performs the task of scaffold decoration and performs a certain activity-driven molecule generation based on preprocessed activity datasets, but requires validation screening by molecular docking. The same activity-data-driven lead optimization model, REINVENT 4 [58], which combines TL, RL, and CL, has improved the efficiency and quality of molecule generation.

Finally, models such as EMPIRE and Scaffold Decorator lack explicit modeling of synthetic pathways (such as reaction conditions and yields). Insufficient feasibility prediction leads to the fact that the generated molecules often cannot enter the experimental validation stage because of high synthetic difficulty. Models that incorporate chemical reaction rules into the preprocessing process, such as LibINVENT [56], constrain the synthetic feasibility of generated molecules through predefined reaction types, thereby improving the synthetic feasibility of molecules.

## Conclusion and prospects

In response to the scarcity of application cases of molecular generation models in the field of NPs, this paper integrates limited research cases and combines successful practices in the field of synthetic drug molecules to verify their cross-domain applicability and technical feasibility. For example, Hao et al. successfully obtained compounds with high affinity and significant antiviral activity by targeted optimization of the side chains of phenanthridine alkaloids based on the DeepFrag model, confirming the efficiency of AIDD in the targeted modification of NPDs [84]. Further analysis shows that molecular generation models have multi-dimensional technical potential in optimizing the structures of NPs: **a) Efficient exploration of chemical space**: for example, Scaffold Decorator generates more than 90,000 virtual molecules from predefined modification sites of the scaffolds, significantly improving the efficiency of lead compound discovery [89]; **b) Design of novel scaffolds**: for example, GraphGMVAE generates novel molecules from the known JAK1 inhibitor upadacitinib, 97.9% of which contain novel scaffolds, breaking through patent limitations and providing a new paradigm for NP-based scaffold innovation [71]; **c) Target-driven precise optimization**: for example, SAMOA combines the 3D structure of ATM kinase with ligand similarity to generate a highly selective inhibitor compound **10r** (IC_50_ = 1 nM), which shows no significant off-target effects on other 103 kinases [96]; **d) Activity-oriented molecular generation**: for example, SyntaLinker integrates the TBK1 kinase inhibitor dataset through TL to generate the target-selective inhibitor **7l**, which has an IC_50_ value of 22.4 nM for TBK1 inhibition. It offers a reference for applying activity-data-driven models to NPs with unknown mechanisms of action [99]. These cases demonstrate the feasibility and applicability of molecular generation models for optimizing the structures of natural products. Although progress has been made, contemporary research continues to depend significantly on validation experiences derived from synthetic drug development. Therefore, there is an urgent need to develop NP-specific generation strategies and evaluation systems.

The application of target-interaction-driven models in NPs is limited by the following: **a) High data acquisition costs**: large amounts of high-quality protein–ligand complex data (e.g., SPR and X-ray crystallography) or from limited public databases (e.g., BindingDB) are required. However, NP-protein complex structures are extremely scarce and expensive to validate experimentally [100]; **b) Excessive target dependence**: the predictive ability of a model is highly dependent on the structural and functional information of known targets. If the conformational changes in the target protein (e.g., metastable effects) are not adequately characterized, or if there are multi-target synergistic effects, the model may fail to accurately predict the binding affinity; **c) Limited generalization**: the model generalizes poorly to new or cross-species targets; **d) Poor dynamic adaptation**: target proteins may change their binding properties owing to the cellular microenvironment (e.g., pH and ion concentration) or post-translational modifications (e.g., phosphorylation). Static datasets are difficult to cover these dynamic changes, leading to prediction deviations [101].

The application limitations of activity-data-driven models in NPs include the following: **a) Data bias and noise sensitivity**: the over-representation of positive molecules and the lack of negative results in the activity dataset lead to model overfitting of the known chemical space and limited diversity of generated molecules. Meanwhile, the differences in the experimental conditions (e.g., pH and temperature) of the activity data reported in different studies are not standardized, which affects the model generalization performance of the prediction model. **b) Lack of mechanistic interpretability**: the model correlates structure and activity through statistical regularities but cannot to clarify specific molecular mechanisms of action (e.g., target binding mode and signaling pathway regulation), limiting its application in mechanism-oriented optimization. **c) Insufficient chemical space coverage**: existing activity datasets mainly focus on specific compound categories (e.g., small molecules), with insufficient characterization of complex natural products (e.g., macrolides and polyketides) and novel synthetic molecules, leading to undesirable model performance in the prediction of new chemical entities [102, 103].

Currently, molecular generation models face several common challenges in NPs applications, including: **a) High computational resource dependency**: a large number of computational resources are required for training, especially for deep learning models (e.g., transformer-based models), which restricts their application in resource-limited scenarios; **b) Inadequate modeling of complex systems**: neither target-interaction nor activity-data-driven models can fully simulate the complexity of real biological systems (e.g., gene interaction networks and metabolic pathway regulation), leading to discrepancies between predictions and in-vivo effects [104]; **c) Explainability and ethical risks**: models generate molecules through implicit associations, and their structure–activity relationships lack transparent resolution. They may produce results that do not align with biological common sense or ethical guidelines (e.g., toxic molecules), necessitating expert knowledge validation.

The intelligent transformation of NPs structural modification faces a historical opportunity, but requires systematic breakthroughs in data, algorithms, and technology. NPs-based molecular generation is characterized by small sample data and most of the targets have not been elucidated. **a) Sample learning and data augmentation**: for example, constructing virtual molecular libraries based on reaction rule-driven molecular modularization [105] or fragmentation [55, 56] to generate chemically reasonable new molecules from limited data; Also, using pre-trained models trained on general molecular libraries (like ZINC and ChEMBL), and fine-tuning them for NPs datasets through TL [58, 77], and combining with an active learning strategy [106], to prioritize the labeling of high-potential molecules and significantly reduce the cost of experimental validation. **b) Dynamic interaction modeling and multi-modal fusion**: for example, introducing equivariant graph neural network and adaptive molecular dynamics to simulate the effect of target allosteric effects on binding stability [107–109]; Constructing “structure–activity-pathway” multi-level prediction models to integrate target interaction networks, transcriptomics and metabolomics data to analyze the multi-target synergistic mechanism [110]. **c) Lightweight model architecture**: large parametric models are compressed into lightweight versions by knowledge distillation techniques to reduce computational resource consumption while maintaining generation performance [111, 112]. **d) Closed-loop automation systems**: the automated synthesis platform integrates inverse synthesis analysis and automated synthesis platform [113, 114], which has been applied to synthesize compounds (e.g. small molecule drugs). In the future, to solve the structural complexity and build NPs-specific synthetic database for algorithm optimization, deep learning molecular design, automated synthesis, biosynthesis pathway, modular reactor, and high-throughput activity detection should be integrated to build a full-process system of “virtual design → robotic synthesis → experimental feedback” and realize the closed-loop of generation-synthesis-verification of NPs synthesis and structural modification. **e) NPs Databases:** existing NPs databases (for example, NPASS, SuperNatural 3.0, LOTUS, and COCONUT) have formed a complete system in chemical structure analysis and physicochemical property characterization. However, they have limitations such as single-dimensional biological activity data, missing dynamic synthetic pathways, and annotation limitations. Future databases need to be upgraded to intelligent predictive platforms by integrating multimodal data and enabling real-time updates (blockchain tracking) and embedding AI toolchains to transform repositories into intelligent synthetic activity prediction centers.

AIDD is promoting the structural modification of NPs from “trial-and-error optimization” to “data-driven rational design”. Future efforts in lightweight architectures, multi-modal fusion, dynamic modeling, and closed-loop automation may overcome key challenges, such as data scarcity, low synthetic feasibility, and multi-objective conflicts, and promote the efficient transformation of NPs from “chemical entities” to “clinical drugs”.

## Data Availability

All the data and materials provided in the manuscript are obtained from included references and available upon request.
